# The potential of Second Life for university counseling: a comparative approach examining media features and counseling problems

**DOI:** 10.1186/s41039-017-0064-6

**Published:** 2017-12-13

**Authors:** Fu-Yun Yu, Hsiao-Ting Hsieh, Ben Chang

**Affiliations:** 1 Institute of Education, National Cheng Kung University, No.1, University Road, Tainan City, 701 Taiwan, Republic of China; 2 Center for Teacher Education/Graduate Institute of Learning and Instruction, National Central University, Taoyuan City, Taiwan, Republic of China

**Keywords:** Computer-mediated communication, Face-to-face communication, Media features, Preferability, Second Life, University counseling

## Abstract

This study explored the potential of Second Life (SL), a popular multi-user virtual online environment, for university counseling. University students (*n* = 312) were asked to evaluate three counseling channels (i.e., traditional face-to-face, internet, and SL) based on a range of media features deemed relevant and important to counseling and to assess their relative preferability when faced with different counseling problems. For the purposes of this study, counseling rooms with different styles were built, virtual counselors with different genders, ages, and styles were created, and short video recordings of scripted counseling sessions were produced in SL. For the media feature comparisons, the collected data were analyzed by the multivariate analysis of variance, followed by the analysis of variance and post hoc comparisons when significant differences were found, whereas for counseling problem comparisons, repeated-measure analysis of variance and post hoc comparisons were used. The results for the media feature comparisons showed that SL counseling significantly out-rated traditional counseling in all of the examined media features, with the exception of the interactivity dimension. Additionally, while SL and internet counseling were both perceived as significantly better than traditional counseling in areas that are unique to computer-mediated communications, including anonymity, convenience, and flexibility with regard to time and space, as well as privacy of the counseling site, SL was perceived as distinctly superior to internet counseling in five areas due to its unique affordances, including the choice of appearance, choice of counselors, interactivity, diversity of counseling sites, and availability of counseling object dimensions. Furthermore, traditional counseling was regarded as better able to support more fluent and versatile interaction between the counselor and client than the other two computer-mediated channels. As for the results of counseling problem comparisons, SL was rated as least preferred for six out of the seven counseling problems (except for gender identity issues), despite its media affordances. Suggestions for practitioners and future research are provided based on the current findings.

**Electronic supplementary material:**

The online version of this article (10.1186/s41039-017-0064-6) contains supplementary material, which is available to authorized users.

## Introduction

Counseling aims at helping people to address emotional, educational, social, vocational, and health-related issues and to promote personal and interpersonal understanding, growth, and functioning in an atmosphere of privacy, confidentiality, trust, support, comfort, and respect, as facilitated by professional counselors (Gladding 2004; Sheppard 2004). Through cognitive, affective, behavioral, or systemic interventions and directed by mental health, psychological, or human development principles (National Board for Certified Counselors (NBCC) 2016), counselors work by helping their clients to discuss challenging situations, release negative feelings and thoughts, analyze themselves and their surroundings, and explore viable coping mechanisms without the fear of being judged, criticized, or ridiculed (American Counseling Association (ACA) 2005; British Association for Counselling (BAC) 1986).

Nowadays, there are different approaches to counseling, which traditionally has taken place in a face-to-face fashion (i.e., in-person counseling). However, with the fast development and spread of computers and networked technologies, internet counseling (i.e., counseling carried out at a distance using computer-mediated text or voice-based tools) is increasingly being used as an alternative to traditional in-person services (Cook and Doyle 2002; Kato et al. 2011). While anonymity and convenience are the advantages of internet counseling, the absence of non-verbal information, such as the client’s body language and facial expressions during the encounter, has raised some concerns about this approach (DeGuzman and Ross 1999; Leibert et al. 2006). With features like avatar creation for individual on-screen representation and the option to use more than 40 built-in actions or movements to display emotional states (Hew and Cheung 2010; Liu 2008), Second Life (hereinafter termed SL) may be able to overcome some of the weaknesses associated with internet counseling. In fact, SL is the most widely used virtual world for higher education purposes (Inman et al. 2010) and is also the most cited multi-user virtual environment in the educational literature (deNoyelles and Seo 2012).

While a large number of publications on SL are available (Cheng 2014; Inman et al. 2010; Wang and Burton 2013), there is still a need for research to examine the unique attributes and strengths of immersive virtual worlds for use in various settings (Dede 2009; Hassan et al. 2016; Hew and Cheung 2010; Witt et al. 2016). In view of the fact that most university counseling services in Taiwan suffer from insufficient counseling space and staff (Ministry of Education, Taiwan 2012) to meet the increasing demand for psychological and counseling assistance of their constituents (John Tung Foundation 2012), the primary goal of this study is to examine whether SL is a viable channel for university counseling. Specifically, SL’s potential was compared against two widely used counseling channels available in universities, based on a set of media features identified as relevant and important for counseling services. In particular, the current study examines the following two research questions:

1. Are there significant differences among the three counseling platforms (i.e., traditional, internet, and SL counseling) with regard to media features, as perceived by university students?
2. If so, in what dimension(s) do these channels differ, and how?

Additionally, with the affordances and limitations associated with different counseling channels, whether university students have a preference for one channel over the others when facing different problems regarding personal growth, wellness, and career development (e.g., life transition, relationship issues, academic problems, interpersonal communication, economic hardship, family conflict, and gender identity) serves as the second issue examined in this study. Two questions are considered in this respect:

1. Is there a significant interaction between counseling platforms and problem types? That is, do university students’ preferences for counseling channels differ dependent on the specific problems encountered in college life?
2. If so, for which problem(s) do these channels differ, and how?

While examining the applications of SL to counseling services in university settings and students’ perspectives on this counseling channel have both empirical and practical importance, the proposed research questions bear special significance for university counseling in Taiwan. First of all, according to the results of a survey done by the John Tung Foundation (2012), about one out of five university students (18.7%) suffer from noticeable depression-associated symptoms and problems that are in need of professional assistance. In addition, according to statistics from the Suicide Prevention Center of Taiwan Suicide Prevention Society (2016), the percentage of deaths from suicide has risen every year since 2000 for the 15~24 age group. These statistics point to the importance of providing accessible counseling service to university students. As noted above, most university counseling services in Taiwan suffer from a severe lack of facilities (Ministry of Education, Taiwan 2012), and thus, how to provide the services needed via alternative channels demands serious attention. In view of the facts that the current generation of learners are so-called digital natives (Prensky 2001), that the 20~29 age group (i.e., university and graduate students in Taiwan) accounts for the largest percentage of the staggering 12.8 million online gaming population out of the total population of 23 million in Taiwan (43.9%), and that among the various genres of online games those based on role-playing are the most popular for desktop and web-based gaming (Fan 2016), then it is of clear interest to examine whether SL, with its role-playing interactive environment, appeals to Taiwanese university students as a viable counseling channel.

In the following sections, the features, applications, and reported benefits of SL are reviewed before moving on to a description of the study.

### Literature review

#### Distinct features of Second Life (SL)

SL is a computer-generated three-dimensional virtual world (Conklin 2007). The simulated environment highlights a participatory culture and is mainly built and maintained by its virtual residents (Santo 2009). To participate, users must first create avatars as their personas in the virtual world by selecting from a number of standard ones, and, if they wish to, they can then personalize these by changing their appearance in a wide variety of ways (e.g., gender, body size, hair color and style, facial features, clothing, and accessories) (Wang and Burton 2013).

With the aid of the SL viewer, users can move around the virtual space by walking, running, flying, or teleporting to other locations and interact with other avatars via text or speech in real time, as well as attend live events (Inman et al. 2010; Wang and Burton 2013). In addition to text and voice chat for public communication and instant messaging for talking to someone in private, non-verbal cues can be displayed to convey personal likes and dislikes, as well as more detailed information about one’s current emotional states, by controlling the avatar’s postures, gestures, and movements (Hew and Cheung 2010; Witt et al. 2016).

#### Pedagogical and therapeutic uses of SL

Due to the distinct features outlined above, SL has been widely used in education and training to provide communication and experiential learning via simulation and modeling in a number of domains, including languages, health and medicine, special education, psychology, graphic design and visual arts, business, social work, architecture, urban planning, anthropology, sociology, environmental education, the military, geography, politics, economics, mathematics, biology, physics, and computing (Hew and Cheung 2010; Inman et al. 2010; Reinsmith-jones et al. 2015; Santo 2009; Wang and Burton 2013; Yuen et al. 2013). Worldwide, more than 150 colleges and universities have virtual facilities in SL, such as virtual campuses with offices, classrooms, and conference venues for live seminars, lectures, and office-hour sessions, all operated to support scholarly exchanges of knowledge and learning without the inconvenience and expense involved in real travel and facilities (Cheng 2014; Jennings and Collins 2007; New Media Consortium (NMC) 2009; Walker and Rockinson-Szapkiw 2009; Wang and Burton 2013). Virtual art museums, science labs, performance centers, and libraries have also been created in SL to extend educational opportunities to those who would not otherwise have access to such facilities (Goodband et al. 2008; Jennings and Collins 2007; Turkay 2012).

Skill training sessions using role-play are increasingly used in SL to offer students hands-on experience in a safe, economically feasible, and virtually real environment (Gregory and Masters 2012; Hudson and Degast-Kennedy 2009; Jamaludin et al. 2009; Walker and Rockinson-Szapkiw 2009). With its powerful simulations and interactive capability, students immersed in SL can experience reenacted or mock scenarios, such as schizophrenic hallucinations (Sherwin 2007), economic collapses (Bloomfield 2008), medical practices (Goral 2008; Lowes et al. 2012), customs and immigration procedures (Hudson and Degast-Kennedy 2009), and natural disasters (Foster 2007), without the dangers or logistical problems that would arise in real-world settings.

Moreover, SL has been used for clinicians or counselors to conduct treatments or therapies or as a supplement to in-person sessions, with more than 100 groups and institutes offering mental health services through SL in the USA. By creating scenes visualizing real-life situations, SL enables clients to experience emotions and practice social skills before putting the learned skills to good use in real life (Russ 2012; Witt et al. 2016). Also, with its capability of creating and modifying the intensity of experienced stimuli in a virtually simulated world for clients with different psychological problems (e.g., phobias, anxiety disorders, eating disorders, and posttraumatic stress disorders) (Gorini and Riva 2008; Parsons and Rizzo 2008; Riva et al. 2007), SL has been reported to be successful in treating some patients. For instance, research by Yuen et al. (2013) experimented with an acceptance-based exposure treatment via SL for patients with generalized social anxiety disorders and found comparable improvements for SL treatments and in-person sessions.

#### Reported benefits of SL

With its rich media environment, on-screen avatars, and multiple communication options, SL has the potential to address the problem of social isolation that is frequently associated with online learning (Caprotti and Seppälä 2007; Schultze and Leahy 2009). In addition, the ability for a user to interact with objects and the world around him or her further enables SL to support situated learning in a manageable way (Hew and Cheung 2010). In summary, the reported benefits associated with the use of SL in education, training, and counseling include:

- Enhanced social presence (Campos-Castillo 2012; Schultze and Leahy 2009),
- Facilitated communication (Hew and Cheung 2010; Cheng 2014),
- Enhanced understanding of learned concepts, and integrated theories and concepts (Caprotti and Seppälä 2007; Houser et al. 2011),
- Active participation and engagement with course content (Lan et al. 2013; Wang and Burton 2013),
- Improved argumentative and critical thinking skills in essay writing (Jamaludin et al. 2009),
- Elevated learning motivation and engagement (Wehner et al. 2011),
- Better formation of sense of community (Wang and Burton 2013),
- Improved egocentric and exocentric perspective-taking (Dede 2009),
- Alleviated concerns over negative evaluations or social stigma during in-person interactions (Olfson et al. 2000),
- The creation of experiences that would be problematic or impossible in real life (Witt et al. 2016),
- Empathetic understanding of mental illnesses (Sherwin 2007),
- Enhanced interview skills (Hudson and Degast-Kennedy 2009) or counseling skills (Walker and Rockinson-Szapkiw 2009; Witt et al. 2016),
- Improved communication skills (Lowes et al. 2012), and
- Better management of emergency situations (Foster 2007).

## Methods

### The purpose and theoretical framework of the study

Although SL is being used extensively in education, training, and psychological therapies, its applications to counseling services in university settings and students’ perspectives on this counseling channel remain to be investigated. To this end, this study examined the relative strengths and weaknesses of SL in comparison to the two most widely adopted counseling service channels (i.e., traditional and internet counseling), along with a range of media features deemed essential for university counseling as well as undergraduates’ preferences for different channels when faced with various psychological problems.

For the purpose of this work, two models which have been extensively studied and validated in many domains to explain the adoption of innovative technologies are used as the theoretical framework of the study—Rogers’s diffusion of innovations theory (Rogers 2003) and Davis’s technology acceptance model (TAM) (Davis 1989).

Briefly explained, predictors affecting users’ adoption behavior and the extent of innovation diffusion are highlighted in Rogers’s diffusion of innovations theory (Rogers 2003). As the factor of relative advantages (that is, the ratio of expected advantages and resources demanded as a result of adopting an innovation; the degree to which an innovation is perceived as better than the other options) has been reported as the strongest predictor (Rogers 2003), all comparisons along media features and counseling problems in this study are done in relative terms (i.e., compared against one another). Moreover, as proposed in TAM, the perceived usefulness and ease of use of a technology affect attitude formation, which in turn impacts the behavioral intention to use and future actual use of the technology, data on the participants’ perceived usefulness of the respective counseling channels for resolving different psychological problems are assessed, following a comparative assessment of the media features of the three counseling channels.

### The definitions of the three counseling channels

Traditional counseling is defined in this study as involving synchronous interaction between the counselor and client using what is seen and heard in person to communicate (National Board for Certified Counselors (NBCC) 2016). Internet counseling, on the other hand, involves the use of computer-mediated communication tools, including e-mail, online chats, bulletin board services, or instant messaging, to allow communication asynchronously and synchronously at a distance between the counselor and client when circumstances make this approach necessary or convenient (Cook and Doyle 2002; Lei 1998). Finally, SL counseling involves synchronous interaction between the counselor and the client within counseling spaces built in SL.

### Counseling spaces built in SL

**Fig. 1:**
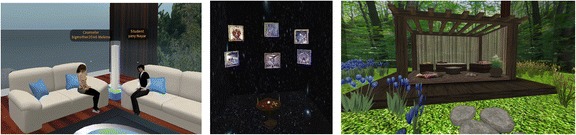
Counseling rooms with different styles: **a.** traditional (left), **b.** numerology (middle), **c.** naturalistic (right)

For the purpose of this study, counseling rooms with different styles were built in SL. A pilot study with 45 university students was carried out prior to the actual study to identify the styles they felt were most appealing. Based on feedback received in the pilot study (see next section), three counseling rooms were constructed—a traditional room, one that used tarot imagery, and one with a more naturalistic feeling. The traditional counseling room was basically modeled on the rooms that are often seen in universities (see Fig. 1a). The counseling room using tarot imagery was developed, as this ties in with Chinese beliefs in predestination, which influence the behavior of many Chinese people (Yang and Lu 2005) (see Fig. 1b). Finally, the counseling room with a naturalistic feeling was designed based on suggestions from the participants in the pilot study (see Fig. 1c).

**Fig. 2:**
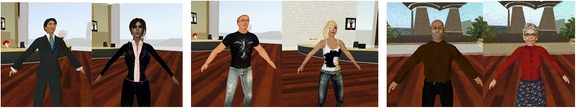
Counselors with different characteristics: **a.** middle-aged counselors with a professional look (left), **b.** young counselors in casual clothing (middle), **c.** elderly counselors in casual clothing (right)

In addition, six counselors with different genders, ages, and styles were created based on feedback from the pilot study: middle-aged male and female counselors with a professional look (Fig. 2a), young male and female counselors dressed casually (Fig. 2b), and elderly male and female counselors, dressed casually (Fig. 2c).

**Fig. 3:**
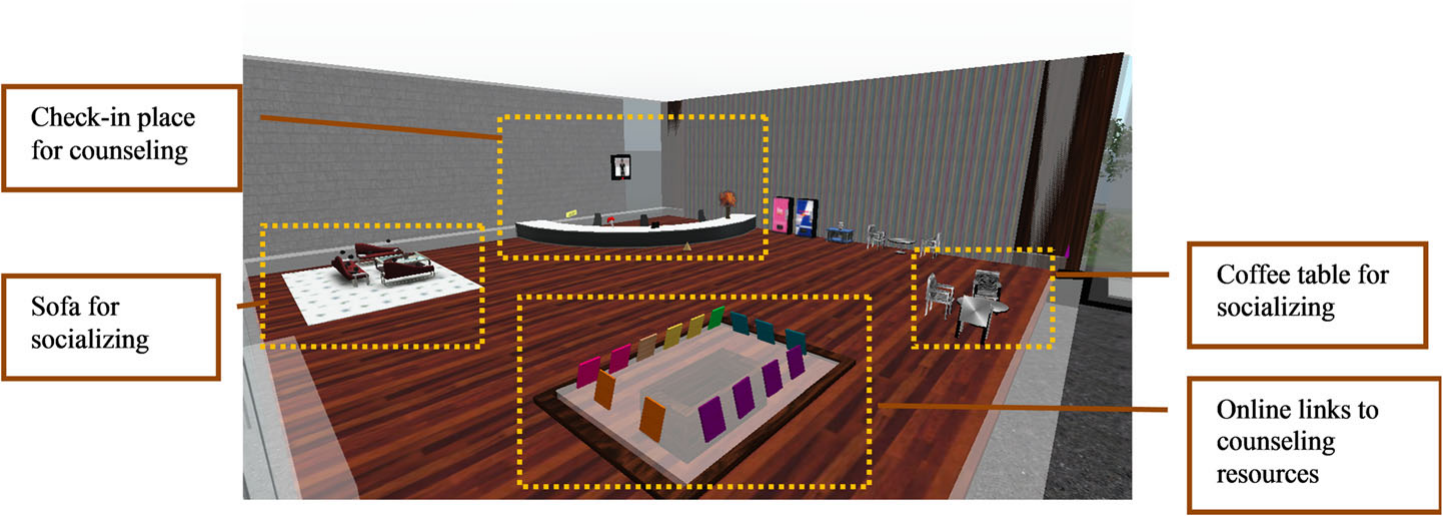
Lobby

A lobby was also created (Fig. 3), with this area reserved for check-in, social interaction, and access to online counseling resources. At check-in, clients can choose which counselor they prefer to have a counseling session with and in which room.

**Fig. 4:**
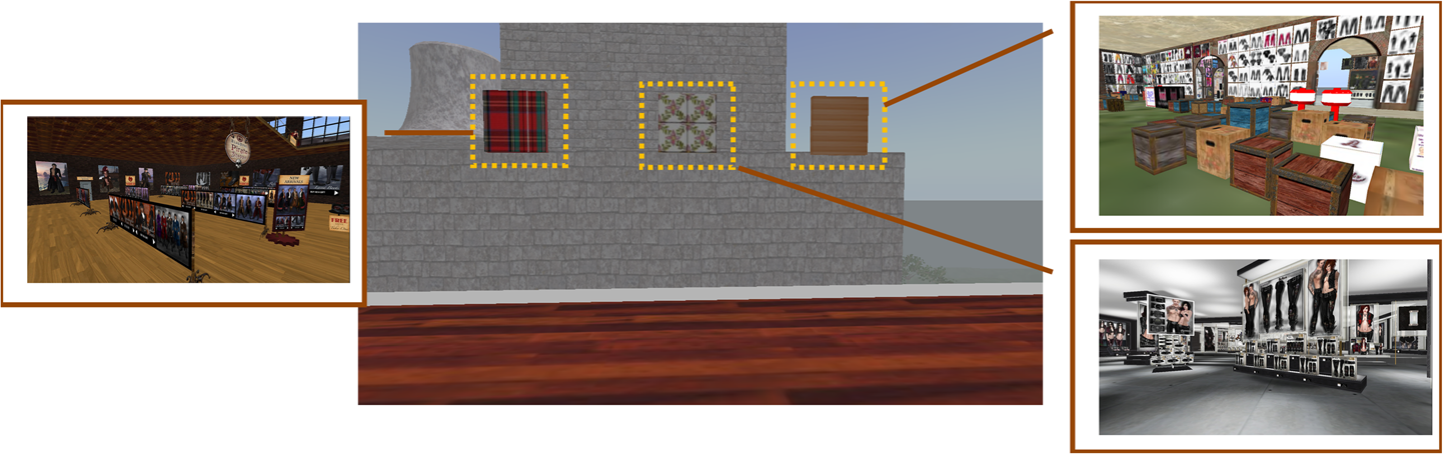
Dressing room with built-in teleports

Finally, to further allow participants to change their appearance, a dressing room was built with links to teleport the client to a few selected spaces where clothing and/or accessories can be purchased (Fig. 4).

### Implementation procedures

Before the actual study, a pilot study was conducted to assess the clarity and comprehensibility of the explanations given to the participants via the three counseling channels and the appeal and appropriateness of the constructed SL spaces. A class of 45 undergraduate students majoring in psychology was recruited. The feedback gathered led to revisions to the implementation procedures, as well as to the spaces constructed in SL. Most importantly, counseling rooms and counselors with different characteristics were created. Moreover, short video recordings of scripted counseling sessions in SL were produced. These video clips allow participants to vicariously experience how counseling is played out in SL and how avatars may interact with others, change their appearance, and move around the constructed spaces, including choosing specific counselors and counseling spaces.

Five classes purposively selected from a public university participated in the actual study (*n* = 312 with 64.7% male). Among these, two were from the College of Social Sciences (*n* = 46); one was from the College of Engineering and Computer Sciences (*n* = 69), and two others were from the general education program (*n* = 197), which enrolled students from all nine colleges of the university where the study took place.

In each of the data collection sessions, a 20-min introductory session on traditional and internet counseling practices and the counseling spaces built in SL by the research group was provided in face-to-face group sessions. In view of SL’s steep learning curve and the participants’ lack of prior experience in SL, the participants were not given an opportunity to interact with others on the platform. Instead, three video clips, each of which deals with different psychological problems, counseling scenarios, and counseling approaches (e.g., analytic psychology, cognitive therapy, humanistic psychology) were shown to the participants, to allow them to observe first-hand what the virtual environment constructed for counseling purposes may look like and how the interaction between the counselor and client may take place in SL.

**Fig. 5:**
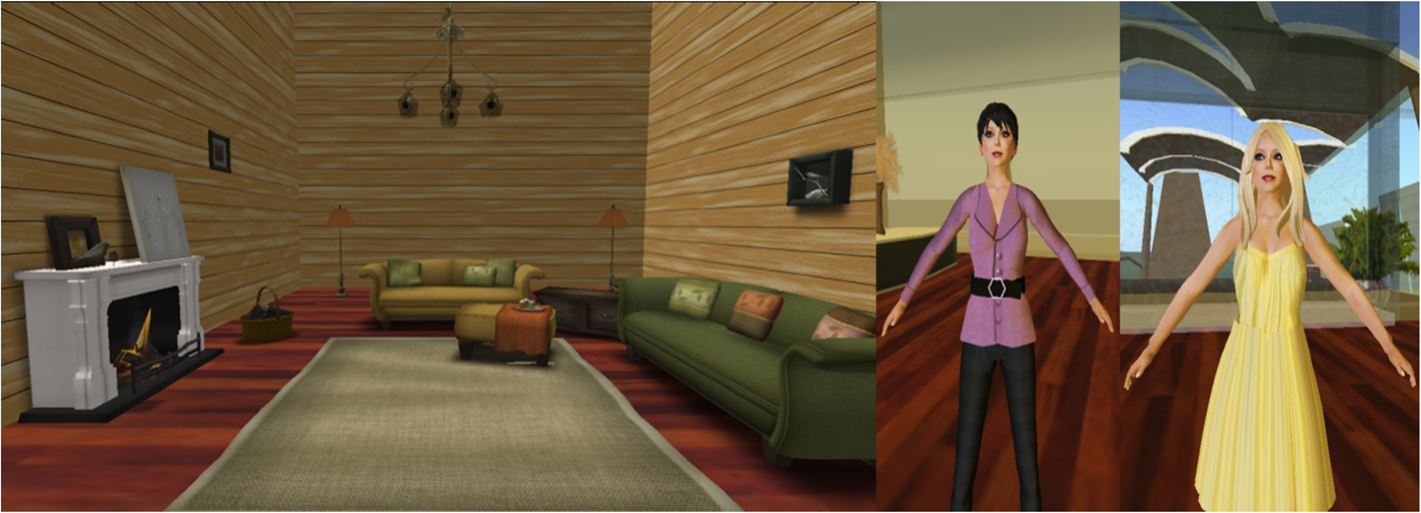
Scripted video clip 1 (counseling session in a living room setting)

The first video involved unfinished business with deceased family members, which, according to Gestalt therapy, can adversely and unexpectedly affect a person’s emotional states, if left unattended to (Corey 2009). This scenario was set in a living room with a professional-looking female counselor and a female client (Fig. 5). The chosen communication mode was oral, with frequent non-verbal messages transmitted by the client, such as sobbing and crossing her arms over her chest.

**Fig. 6:**
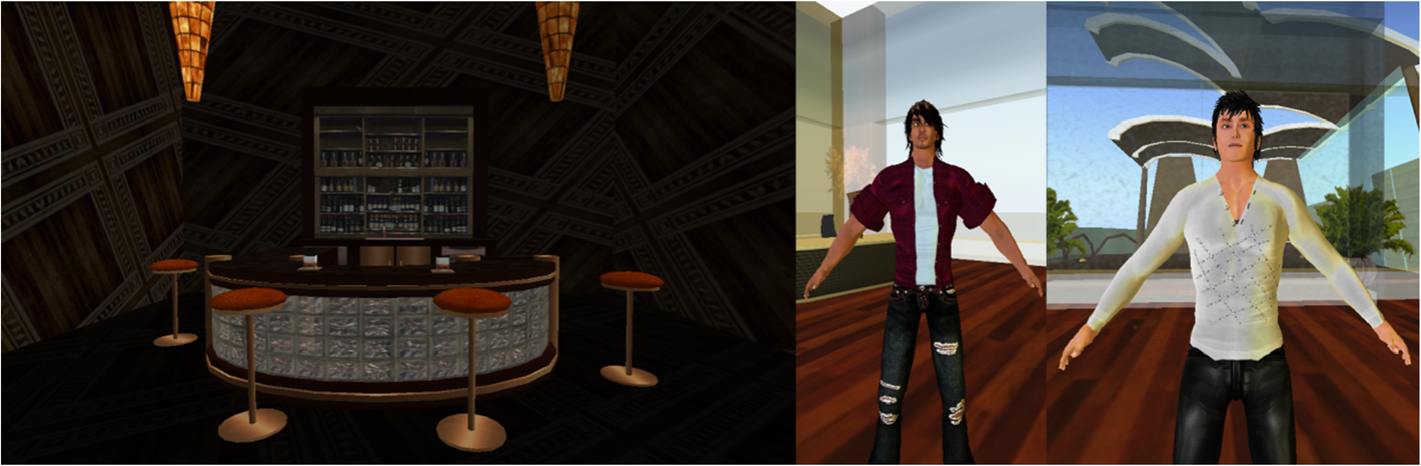
Scripted video clip 2 (counseling session in a bar setting)

As students’ reluctance to seek help was identified as the most difficult problem counselors had with high school and college students dealing with issues related to homosexuality (Liu and Chao 2006), the second video dealt with problems related to sexual identity and orientation. Because of the sensitive nature of this topic, which many people find challenging to discuss in a traditional counseling setting, the communication mode chosen was written text with occasional movements exhibited by the client during the session (such as leaning sideways and crossing their legs). The context designed for this counseling session was in a bar, with both the counselor and client being male (Fig. 6).

**Fig. 7:**
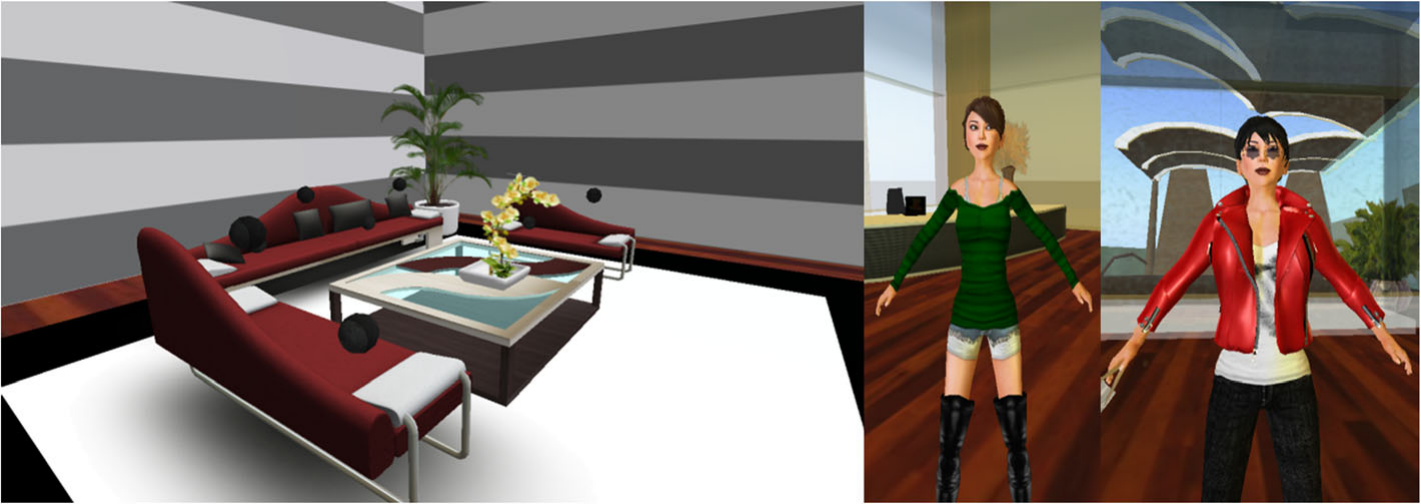
Scripted video clip 3 (counseling session in a modern setting)

The third video clip was about disputes between lovers due to infidelity. The counseling session was conducted in a modern living room, with a young, fashionably dressed female counselor. The client was represented by a standard female avatar, without modifications of any kind (Fig. 7). Both parties interacted orally and activated several built-in movements during the process to express their feelings.

After a 20-min introduction and presentation of the three video clips, the participants were asked to compare and rate the relative strengths and weaknesses of the three counseling channels with regard to the various media features (see the next section for the related definitions and instructions given) and then select the channels they prefer to use for counseling assistance when encountering different psychological problems.

### Instruments

With reference to the literature on the features and limitations of traditional counseling (e.g., insufficient counseling rooms and counselors, the need for the physical presence of the client seeking counseling services, provision of services during limited office hours, clients not protected from being seen when entering the premise to receive counseling) (Cook and Doyle 2002; Lei 1998) and internet counseling (e.g., loss of non-verbal messages) (Cook and Doyle 2002; Leibert et al. 2006), media features deemed likely to have an impact on the processes and outcomes of counseling were summarized and included in the first part of the questionnaire.

Specifically, nine media features were identified and examined in this study, and these were defined and annotated in the questionnaire, as follows:

- Anonymity: the degree to which the client’s identity is protected from being disclosed
- Choice of appearance: the ease with which clients can change their appearance (including gender, hairstyle, and clothing) according to their preferences
- Choice of counselor: the ability of the client to choose who their counselor will be
- Interactivity: the extent to which the interacting parties (i.e., the counselor and client) can send and receive written and oral messages, facial expressions, gestures, and postures, as well as paralanguage elements, such as pitch, volume, and intonation during counseling sessions
- Convenience and flexibility in time: convenience and flexibility with regard to when the counseling session will be held
- Convenience and flexibility in place: convenience and flexibility with regard to where the counseling session will be held
- Privacy of counseling site: the extent to which the clients can be assured that they will not be seen by others when arriving at or leaving a counseling session
- Diversity of counseling sites: the variety of styles and layout among the various counseling rooms offered
- Availability of various objects as aids: the availability and accessibility of various items that are used in traditional counseling sessions, such as crystal balls, toys, and couches

Each of the participants was directed to compare the relative strengths and weaknesses of each of the three counseling channels with regard to each of the nine media features outlined above by rating them on a scale from 1 to 9.

In addition, a number of psychological problems frequently encountered by college students were included in the second part of the questionnaire—life transition, relationship issues, academic problems, interpersonal communication, economic hardship, family conflict, and gender identity. The participants were instructed to first mark the counseling channel(s) they would consider when seeking professional assistance, and then select one from these marked ones as the most preferred channel for each of the problems.

## Results and discussion

### Comparisons of media features

**Fig. 8:**
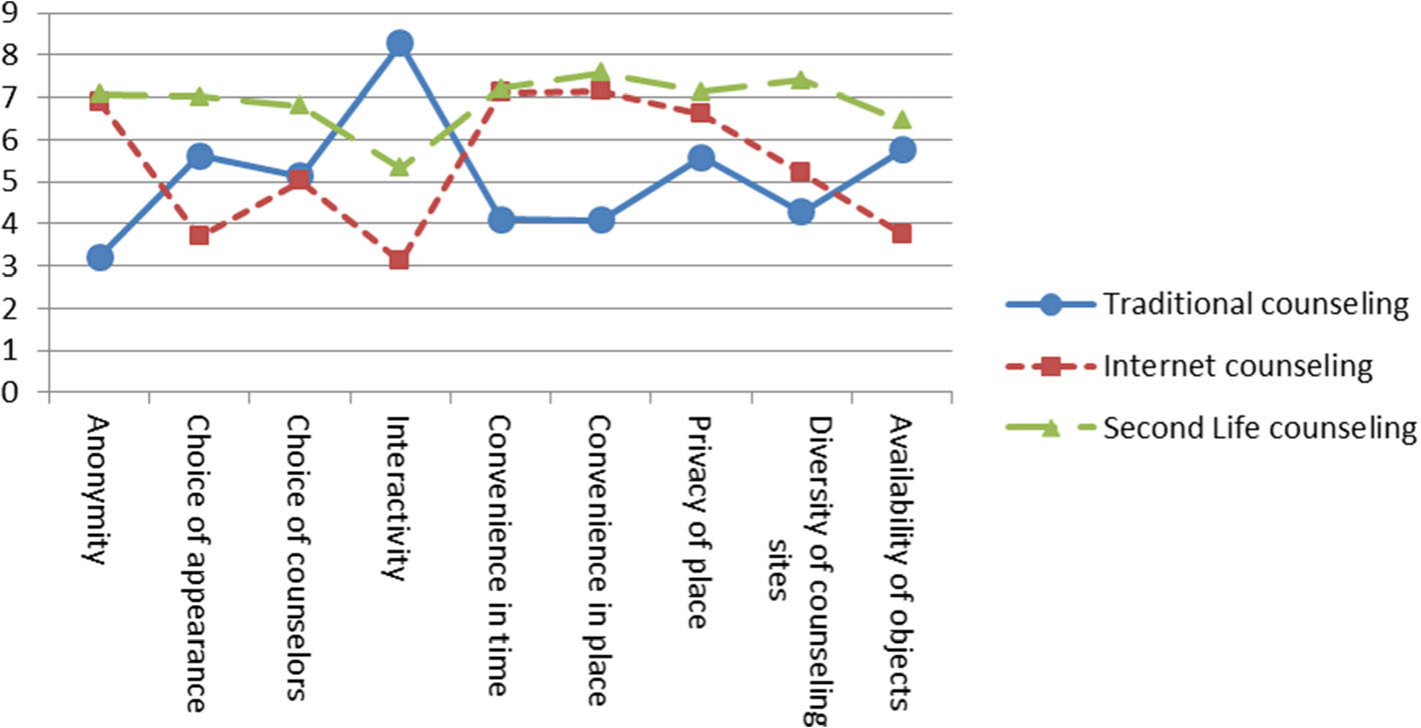
A comparison of the three counseling channels along media features

The means and standard deviations of the participants’ responses for the three counseling channels regarding the nine media features are listed in Table 1 and shown in Fig. 8. It can be seen that of the three counseling channels, SL counseling was perceived the most favorably in all dimensions, with the exception of interactivity, in which case traditional counseling out-rated both SL and internet counseling. In addition, traditional counseling was rated least favorably among the three counseling channels in five out of the nine examined features: anonymity, convenience and flexibility in time, convenience and flexibility in place, privacy of counseling site, and diversity of counseling sites.

**Table 1:** Descriptive statistics for the three counseling channels with regard to the nine media features

Media features examined and rated		Counseling channels		
		Traditional (*n* = 310)	Internet (*n* = 311)	SL (*n* = 311)
Anonymity	*M* (SD)	3.21 (2.19)	6.90 (1.92)	7.07 (1.76)
Choice of appearance	*M* (SD)	5.61 (2.73)	3.70 (2.45)	7.01 (2.09)
Choice of counselors	*M* (SD)	5.13 (2.29)	5.01 (2.31)	6.81 (1.83)
Interactivity	*M* (SD)	8.28 (1.37)	3.11 (1.80)	5.33 (1.90)
Convenience in time	*M* (SD)	4.10 (1.88)	7.11 (1.66)	7.20 (1.50)
Convenience in place	*M* (SD)	4.09 (1.84)	7.15 (1.89)	7.58 (1.49)
Privacy of place	*M* (SD)	5.57 (2.12)	6.60 (2.04)	7.14 (1.79)
Diversity of counseling sites	*M* (SD)	4.27 (2.01)	5.21 (2.23)	7.40 (1.63)
Availability of counseling objects	*M* (SD)	5.76 (2.30)	3.76 (2.24)	6.45 (2.06)

Note: each of the media features was rated on a scale from 1 to 9

Data were further analyzed using the multivariate analysis of variance (MANOVA), followed by the analysis of variance (ANOVA) and post hoc comparisons when significant differences were found. The MANOVA results, using the Roy’s largest root statistic, indicated that the students perceived the three channels as statistically different with regard to media features (*F* = 308.12, *p =* 0.000, partial eta squared = 0.75). The follow-up ANOVAs also indicated that the students perceived the three counseling channels as statistically different in all nine media features (*F* = 382.61, *p =* 0.000, partial eta squared = 0.45 for anonymity; *F* = 144.80, *p =* 0.000, partial eta squared = 0.24 for choice of appearance; *F* = 68.11, *p =* 0.000, partial eta squared = 0.13 for choice of counselors; *F* = 719.07, *p =* 0.000, partial eta squared = 0.61 for interactivity; *F* = 338.73, *p =* 0.000, partial eta squared = 0.42 for convenience and flexibility in time; *F* = 368.44, *p =* 0.000, partial eta squared = 0.44 for convenience and flexibility in place; *F* = 49.52, *p =* 0.000, partial eta squared = 0.10 for privacy of place; *F* = 205.61, *p =* 0.000, partial eta squared = 0.31 for diversity of counseling sites; *F* = 125.49, *p =* 0.000, partial eta squared = 0.21 for availability of objects).

As shown in Table 2, post hoc comparisons with Bonferroni corrections further showed that SL counseling was perceived as significantly better than traditional counseling in all dimensions, with the exception of interactivity. Moreover, internet and SL counseling were perceived as significantly superior to traditional counseling with regard to anonymity, convenience and flexibility in time, convenience and flexibility in space, privacy of counseling site, and diversity of counseling sites. Furthermore, SL counseling was perceived as significantly better than internet counseling in the following five features: choice of appearance, choice of counselors, interactivity, diversity of counseling sites, and availability of counseling objects. Finally, traditional counseling was perceived as significantly better than internet counseling in three areas: choice of appearance, interactivity between interacting parties, and availability of counseling objects as aids.

**Table 2:** Comparisons of the three counseling channels with regard to media features

	Comparison		Mean differences (I-J)	SE	*p* value	95% confidence interval	
	Channel (I)	Channel (J)				Lower	Upper
Anonymity	Traditional	Internet	− 3.69*	.16	.000	− 4.25	− 3.13
		SL	− 3.86*	.16	.000	− 4.45	− 3.30
	Internet	SL	− 0.17	.16	.557	− 0.73	0.39
Choice of appearance	Traditional	Internet	1.91*	.20	.000	1.21	2.61
		SL	− 1.41*	.20	.000	− 2.10	− 0.71
	Internet	SL	− 3.32*	.20	.000	− 4.01	− 2.62
Choice of counselors	Traditional	Internet	0.11	.17	.808	− 0.50	0.73
		SL	− 1.69*	.17	.000	− 2.30	− 1.07
	Internet	SL	− 1.80*	.17	.000	− 2.42	− 1.19
Interactivity	Traditional	Internet	5.17*	.14	.000	4.68	5.65
		SL	2.95*	.14	.000	2.46	3.44
	Internet	SL	− 2.22*	.14	.000	− 2.70	− 1.73
Convenience in time	Traditional	Internet	− 3.01*	.14	.000	− 3.49	− 2.53
		SL	− 3.10*	.14	.000	− 3.58	− 2.61
	Internet	SL	− 0.08	.14	.826	− 0.57	.40
Convenience in place	Traditional	Internet	− 3.07*	.14	.000	− 3.57	− 2.57
		SL	− 3.49*	.14	.000	−3.99	−2.99
	Internet	SL	− 0.42	.14	.011	− 0.92	.078
Privacy of counseling site	Traditional	Internet	− 1.02*	.16	.000	− 1.59	− 4.53
		SL	− 1.56*	.16	.000	− 2.13	− 1.00
	Internet	SL	− .54	.16	.003	− 1.11	.02
Diversity of counseling sites	Traditional	Internet	− 0.94*	.16	.000	− 1.50	− 0.38
		SL	− 3.13*	.16	.000	− 3.69	− 2.56
	Internet	SL	− 2.19*	.16	.000	− 2.75	− 1.63
Availability of counseling objects	Traditional	Internet	2.01*	.18	.000	1.38	2.63
		SL	− 0.69*	.18	.001	− 1.31	0-.06
	Internet	SL	− 2.69*	.18	.000	− 3.32	− 2.06

Note: *p* values have been adjusted with the Bonferroni method

*p < .05

### Comparisons of counseling problems

**Fig. 9:**
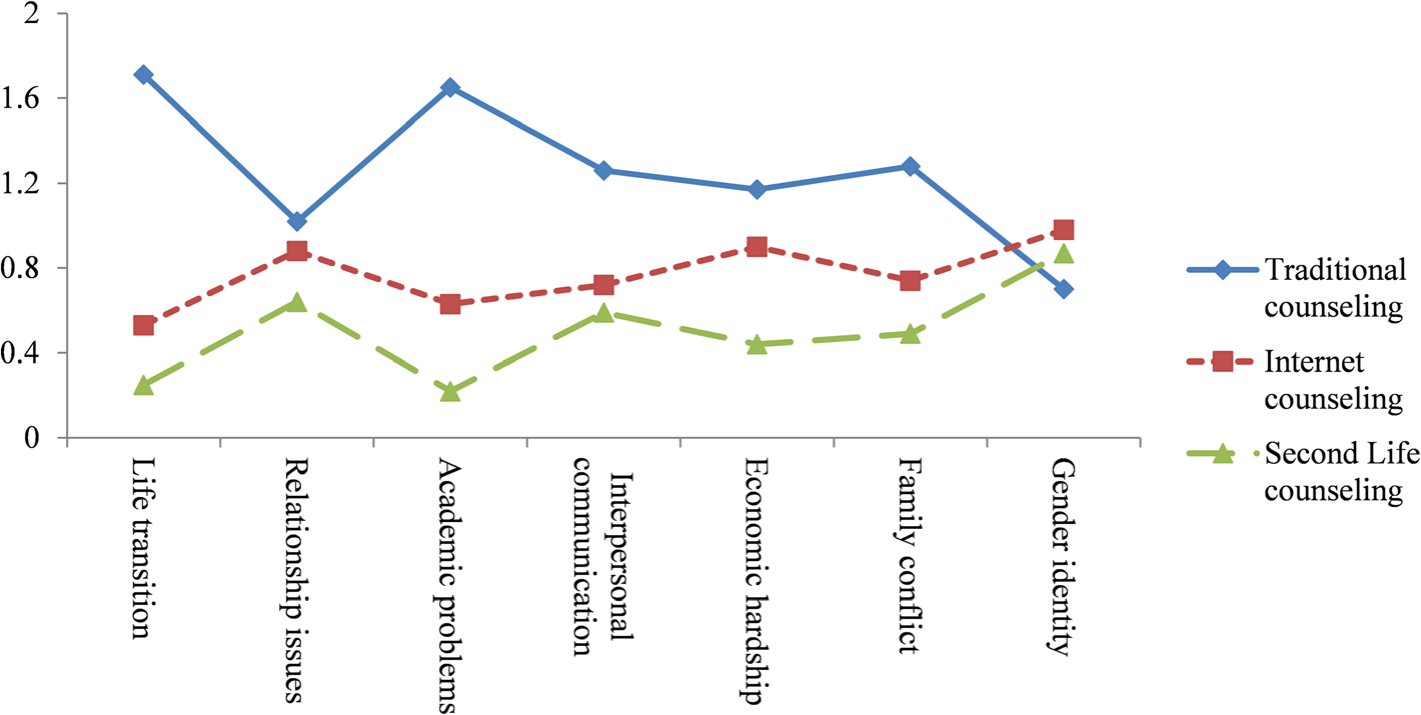
Comparisons of the three counseling channels along with counseling problems

The participants’ responses were recoded prior to analysis. For a given counseling problem, a counseling channel was scored 1 if it was selected, scored 2 if it was selected and marked as their most preferred channel when seeking professional assistance for the targeted psychological problem, and scored 0 if it was not selected. The means and standard deviations of the rated preferability of the three counseling channels with respect to the respective counseling problems are listed in Table 3 and shown in Fig. 9. The results showed that traditional counseling was rated as most favorable and SL counseling as least with respect to all counseling problems, except for gender identity issues, for which internet counseling was rated as most favorable and traditional counseling as least.

**Table 3:** Descriptive statistics of respondents’ preference for the three counseling channels with regard to seven counseling problems

Counseling problems	Counseling channels					
	Traditional (*N* = 312)		Internet (*N* = 312)		SL (*N* = 312)	
	*M* (SD)	%	*M* (SD)	%	*M* (SD)	%
Life transition	1.71 (0.61)	79.49	0.53 (0.73)	14.10	0.25 (0.52)	4.17
Relationship issues	1.02 (0.92)	42.95	0.88 (0.86)	31.09	0.64 (0.83)	22.76
Academic problems	1.65 (0.68)	76.28	0.63 (0.78)	18.27	0.22 (0.48)	2.56
Interpersonal communication	1.26 (0.9)	56.73	0.72 (0.79)	21.15	0.59 (0.79)	18.91
Economic hardship	1.17 (0.9)	50.96	0.9 (0.88)	33.65	0.44 (0.7)	12.18
Family conflict	1.28 (0.89)	57.69	0.74 (0.83)	24.04	0.49 (0.75)	15.38
Gender identity issues	0.7 (0.88)	27.88	0.98 (0.86)	35.90	0.87 (0.88)	33.01

Note: The average scores, *M*, were created by averaging the recorded scores across all the respondents (0 = unselected, 1 = selected, 2 = selected and marked as most preferred); the % represents the proportion of the respondents who selected a given channel as most preferred among all the respondents

Data were analyzed using repeated-measure ANOVA and post hoc comparisons. The main effect of counseling channels is significant (*F* = 118.23, *p* = 0.000), as is the interaction between counseling channels and counseling problems (*F* = 55.65, *p* = 0.000), suggesting that the participants’ rated preferability does indeed differ among the three channels, and the differences in the rated counseling channels also varies across counseling problems. The main effect of counseling problems is not significant (*F* = 1.74, *p* = .12), such that the overall ratings for a channel’s counseling do not differ with the counseling problems.

When further looking at the simple main effects of the counseling channels with respect to each problem, all the effects are significant (*F* = 377.85, *p =* 0.000, partial eta squared = .55 for life transition; *F* = 10.88, *p =* 0.000, partial eta squared = .03 for relationship issues; *F* = 297.32, *p =* 0.000, partial eta squared = .49 for academic problems; *F* = 41.86, *p =* 0.000, partial eta squared = .12 for interpersonal communication; *F* = 45.89, *p =* 0.000, partial eta squared = .13 for economic hardship; *F* = 54.91, *p =* 0.000, partial eta squared = .15 for family conflict; *F* = 6.00, *p =* .003, partial eta squared = .02 for gender identity issues).

As shown in Table 4, the results of post hoc comparisons with Bonferroni corrections revealed that traditional counseling was perceived as significantly more preferable than both internet and SL counseling in response to life transition, academic problems, interpersonal communication, economic hardship, and family conflict. Both traditional and internet counseling were considered as more preferable than SL counseling in response to life transition, relationship issues, academic problems, economic hardship, and family conflict. Finally, internet counseling was perceived as more preferable than traditional counseling in response to gender identity issues, but was rated as equally preferable as SL counseling, while SL counseling did not significantly differ from traditional counseling in this regard. These findings suggest that when it comes to their own choice of counseling services, and despite acknowledging SL’s various advantages, the university students perceived SL counseling as much less desirable than traditional counseling for most of their problems.

**Table 4:** Comparisons of the three counseling channels with regard to different counseling problems

	Comparison		Mean differences	SE	*p* value	95% confidence interval	
	Channel (I)	Channel (J)	(I-J)			Lower	Upper
Life transition	Traditional	Internet	1.18*	0.08	.000	0.98	1.38
		SL	1.47*	0.08	.000	1.27	1.67
	Internet	SL	0.29*	0.08	.000	0.08	0.49
Relationship issues	Traditional	Internet	0.14	0.08	.38	− 0.07	0.34
		SL	0.37*	0.08	.000	0.17	0.58
	Internet	SL	0.24*	0.08	.002	0.04	0.44
Academic problems	Traditional	Internet	1.02*	0.08	.000	0.81	1.22
		SL	1.43*	0.08	.000	1.22	1.63
	Internet	SL	0.41*	0.08	.000	0.21	0.61
Interpersonal communication	Traditional	Internet	0.54*	0.08	.000	0.34	0.74
		SL	0.67*	0.08	.000	0.46	0.87
	Internet	SL	0.13	0.08	.10	− 0.07	0.33
Economic hardship	Traditional	Internet	0.27*	0.08	.007	0.07	0.48
		SL	0.73*	0.08	.000	0.53	0.93
	Internet	SL	0.46*	0.08	.000	0.26	0.66
Family conflict	Traditional	Internet	0.55*	0.08	.000	0.34	0.75
		SL	0.79*	0.08	.000	0.59	0.99
	Internet	SL	0.25*	0.08	.000	0.04	0.45
Gender identity	Traditional	Internet	− 0.28*	0.08	.003	− 0.46	− 0.10
		SL	− 0.17	0.08	.17	− 0.34	0.01
	Internet	SL	0.12	0.08	.33	− 0.06	0.29

Note: *p* values have been adjusted with the Bonferroni method

*p < .05

### Significance and important findings of this study

In view of the fact that the majority of universities are not equipped with sufficient facilities and staff to offer the much-needed counseling services for their students (John Tung Foundation 2012; Ministry of Education, Taiwan 2012), this study was carried out to examine whether SL can serve as a viable alternative. With its ability to provide compelling virtual environments and avatars with considerable freedom of action to send facial expressions, gestures, postures, and paralanguage (Cheng 2014; Inman et al. 2010; Hew and Cheung 2010; Liu 2008; Wang and Burton 2013), SL appears to be a promising addition to traditional and internet counseling practices. However, issues regarding how the specific features of SL are perceived by university students and how students would approach different counseling channels when faced with personal issues and problems have not yet been examined empirically. Therefore, the goals of this study were to examine the relative strengths and weaknesses of traditional, internet, and SL counseling in terms of the nine media features of relevance and importance, and the relative preferability of the three channels as assessed by university students when dealing with different psychological problems.

With regard to the first goal of the study (i.e., a comparison of media features), four important findings were obtained. First, in terms of media features, SL significantly out-performed traditional counseling in all of the examined features, with the exception of interactivity. In other words, despite the facts that SL’s distinct features were overall well perceived and well recognized by the students and that the platform has been suggested to enable greater interactivity by allowing users to change their postures, gestures, and movements (Hew and Cheung 2010; Witt et al. 2016), as well as supporting multiple communication modes so that the interacting parties can work with verbal and non-verbal messages to a specific person in private, or to a group in public (Inman et al. 2010; Wang and Burton 2013; Hew and Cheung 2010), the level of interactivity that occurred in SL counseling was still perceived by the participants as more limited and less fluent compared to that achieved with the traditional, face-to-face approach to counseling.

Second, SL counseling was rated similarly to internet counseling in terms of anonymity, convenience and flexibility in time and space, and privacy of the counseling site. In addition, both SL and internet counseling were perceived as significantly better than traditional counseling in all features that are unique to computer-mediated communications.

Third, despite their similarity in many aspects, SL counseling out-rated internet counseling in five areas: choice of appearance, choice of counselors, interactivity, diversity of counseling sites, and availability of counseling objects. As noted, while the internet and SL can both provide convenient services with few constraints on time and place of use, and also offer greater privacy for the client, SL counseling was regarded as being superior in respect to a number of its unique features, including customizable on-screen avatars, a rich media environment supporting tele- and social presence, and multiple communication options (Inman et al. 2010; Hew and Cheung 2010; Schultze and Leahy 2009; Wang and Burton 2013).

Fourth, despite the fact that both internet and SL counseling were perceived to provide better privacy protection and greater convenience with regard to time and place, traditional counseling was regarded as better able to support more fluent and versatile interactions between counselors and clients.

As for the second goal of this study (i.e., different forms of counseling in relation to different counseling problems), there are three important findings. First, in general, the university students preferred traditional counseling to internet or SL counseling when they encountered psychological problems other than gender identity issues. Second, internet counseling, unexpectedly, significantly out-rated SL counseling for five out of the seven identified problems (i.e., life transition, relationship issues, academic problems, economic hardship, and family conflict), but did not significantly differ from SL counseling for interpersonal communication and gender identity issues. Finally, internet counseling was preferable to traditional counseling for gender identity issues.

In sum, although the university students rated SL counseling favorably in terms of media features (e.g., privacy protection and convenience), they expressed least interest in using it for most of the given problems encountered in college life, as compared to traditional or internet counseling. Even for problems that are sensitive and thus require greater privacy (e.g., gender identity issues), SL counseling was not the most  preferred channel when professional counseling assistance was needed.

## Conclusions

This study explored university students’ views of the relative strengths and weaknesses of the three counseling channels (i.e., traditional, internet, and SL counseling) with regard to nine media features and investigated their relative preferability when faced with different counseling problems. The results show conclusively that the university students viewed SL counseling more positively than traditional counseling in all of the media features examined, except the interactivity dimension, and better than internet counseling in areas distinctly unique to SL (e.g., choice of appearance, counselors, counseling sites, and counseling object). However, despite SL’s media affordances, SL counseling was not more desirable than traditional and internet counseling when encountering most of the given problems for the university students who took part in this study.

### Implications, suggestions, and precautions for university counseling services

Based on the findings of this study, the following suggestions for university counseling are provided. First, as SL was least desirable when professional assistance was needed for university students’ educational, relationship, psychological, and financial problems, the current problem of a lack of facilities faced by most university counseling services in Taiwan (Ministry of Education, Taiwan 2012) needs to be resolved by other measures (e.g., funding for more counseling rooms and extended opening hours).

With that said, in light of the facts that there was still a substantial percentage of the respondents who most preferred SL (4.17%~33.01, Table 3) when seeking counseling assistance for different problems and that the current generation of university students are digital natives, it is suggested that practitioners still consider the inclusion of SL as an alternative channel. With this, the increasing demand for psychological and counseling assistance can be better attended to, and made available to university students in need.

Additionally, as asserted by environmental psychologists, contexts and surroundings influence people’s behaviors, thoughts, and feelings to a considerable extent (Bell et al. 2000). People may disclose themselves at different levels of depth and speed when communicating face-to-face or online (Parks and Roberts 1998), and the unique aspects of the client may be revealed in different communication modalities supported by different channels (Fenichel et al. 2002). It is thus suggested that multiple counseling channels should be made available, because these can not only provide university students with increased access to counseling services, but can also allow the counselor to adopt a combined approach to attaining synergistic effects to best satisfy the needs of the current situation, and so deliver an enhanced counseling experience to their clients. For instance, if interactivity is a key concern, especially at the initial stage of counseling, where trust, understanding, and a relationship between the counselor and client need to be established (Corey 2009), traditional counseling may be the best choice. After a couple of sessions, when the trust between the counselor and client has been built up, switching to other channels may then be considered. On the other hand, if anonymity, time, privacy, and geographical distance are compelling considerations in the focal context (for instance, when it comes to gender identity issues), internet and SL counseling may be more viable choices. Alternatively, when a counselor wishes to allow elements of imagination, role-play, and fantasy to take place during the exchange, SL may be the best channel.

Even though the advantageous media features of SL for university counseling were well received by the participants and thus empirically supported in this study, a few words of caution are provided in view of the finding that SL was considered the least preferred channel for most of the counseling problems. Although the reasons why the participants selected one channel over the others were not gathered in this study, the users’ perceptions of the risks and difficulties in using SL may account for the results, as reflected in other studies examining the applications of emerging technologies in educational settings (e.g., Cao et al. 2013), and suggested by the diffusion of innovation theory (Rogers 2003) and TAM (Davis 1989). As noted earlier in this paper, issues regarding whether SL counseling is effective and of good quality, and if it would create difficulties and exert extra demands on the part of the users, both in itself and compared to the other counseling channels, are major factors when it comes to considering whether the adoption and diffusion of this technology should be promoted. As such, it is suggested that the availability and suitability of SL in specific context should take into account the client’s subjective evaluation of their individual level of readiness and dominant needs.

Finally, for university counselors and practitioners, there are a number of barriers to consider before adopting SL for university counseling, including the cost of creating and maintaining the facilities in SL, the steep learning curves and feelings of dizziness that may occur when operating in a 3D virtual space, and the need for computers with powerful graphics capabilities and internet connectivity (Caprotti and Seppälä 2007; Cheng 2014; Hew and Cheung 2010; Russ 2012; Wang and Burton 2013; Walker and Rockinson-Szapkiw 2009).

### Limitations of this study and suggestions for future works

In view of SL’s steep learning curve and the participants’ lack of prior experience of this platform, the present study did not provide the participants with an opportunity to interact with others in the virtual world. The two research questions in this work can thus be examined in future studies by having participants experience SL first-hand.

Moreover, the sample of the study consists of undergraduates, who were purposively selected from different disciplines within one comprehensive university, all of whom were currently taking courses on campus and volunteered to participate. Therefore, the generalizability of the results to other groups (e.g., users at a younger age, with more limited computer abilities, having restricted networked accessibility, and in real need of professional assistance for psychological problems) should be exercised with great care.

In addition, because a user’s comfort level grows with increased exposure to 3D simulated environments (Caprotti and Seppälä 2007; Hew and Cheung 2010), and research has found gender differences in perception and acceptance of technology (Chinyamurindi and Louw 2010; Gefen and Straub 1997; Ma and Yuen 2006; Wang and Wang 2008), to what extent users are able to accept SL counseling may be dependent on individual differences in characteristics related to technology use (such as gender and past experience with 3D virtual worlds). Future research along this line with reference to the technology acceptance model would be fruitful.

Finally, counseling sessions are held between a counselor and a client, and the present study only examined the perceptions of the latter. Future studies involving professional counselors to reveal their perspectives on and intentions to accepting and using SL would be worthwhile to complement the preliminary findings of this work.

## Additional file

Additional file 1: Dataset for the two proposed research questions. (XLSX 68 kb)
